# Machine Perfusion Enables 24-h Preservation of Vascularized Composite Allografts in a Swine Model of Allotransplantation

**DOI:** 10.3389/ti.2024.12338

**Published:** 2024-05-15

**Authors:** Marion Goutard, Pierre Tawa, Yanis Berkane, Alec R. Andrews, Casie A. Pendexter, Reinier J. de Vries, Victor Pozzo, Golda Romano, Hyshem H. Lancia, Irina Filz von Reiterdank, Nicolas Bertheuil, Ivy A. Rosales, Ira Doressa Anne L. How, Mark A. Randolph, Alexandre G. Lellouch, Curtis L. Cetrulo, Korkut Uygun

**Affiliations:** ^1^ Division of Plastic and Reconstructive Surgery, Massachusetts General Hospital, Boston, MA, United States; ^2^ Harvard Medical School, Boston, MA, United States; ^3^ Shriners Children’s Boston, Boston, MA, United States; ^4^ Suivi Immunologique des Thérapeutiques Innovantes Laboratory, INSERM U1236, University of Rennes 1, Rennes, France; ^5^ Department of Plastic, Reconstructive, and Aesthetic Surgery, Centre Hospitalier Universitaire de Rennes, Université de Rennes 1, Rennes, France; ^6^ Center for Engineering in Medicine and Surgery, Massachusetts General Hospital, Boston, MA, United States; ^7^ Department of Surgery, Amsterdam University Medical Centers—Location AMC, University of Amsterdam, Amsterdam, Netherlands; ^8^ University Medical Center Utrecht, Utrecht University, Utrecht, Netherlands; ^9^ Immunopathology Research Laboratory, Center for Transplantation Sciences, Massachusetts General Hospital, Boston, MA, United States; ^10^ Department of Pathology, Harvard Medical School, Boston, MA, United States

**Keywords:** vascularized composite allotransplantation, machine perfusion, organ preservation, ischemia-reperfusion injuries, VCA

## Abstract

The current gold standard for preserving vascularized composite allografts (VCA) is 4°C static cold storage (SCS), albeit muscle vulnerability to ischemia can be described as early as after 2 h of SCS. Alternatively, machine perfusion (MP) is growing in the world of organ preservation. Herein, we investigated the outcomes of oxygenated acellular subnormothermic machine perfusion (SNMP) for 24-h VCA preservation before allotransplantation in a swine model. Six partial hindlimbs were procured on adult pigs and preserved *ex vivo* for 24 h with either SNMP (*n* = 3) or SCS (*n* = 3) before heterotopic allotransplantation. Recipient animals received immunosuppression and were followed up for 14 days. Clinical monitoring was carried out twice daily, and graft biopsies and blood samples were regularly collected. Two blinded pathologists assessed skin and muscle samples. Overall survival was higher in the SNMP group. Early euthanasia of 2 animals in the SCS group was linked to significant graft degeneration. Analyses of the grafts showed massive muscle degeneration in the SCS group and a normal aspect in the SNMP group 2 weeks after allotransplantation. Therefore, this 24-h SNMP protocol using a modified Steen solution generated better clinical and histological outcomes in allotransplantation when compared to time-matched SCS.

## Graphical Abstract

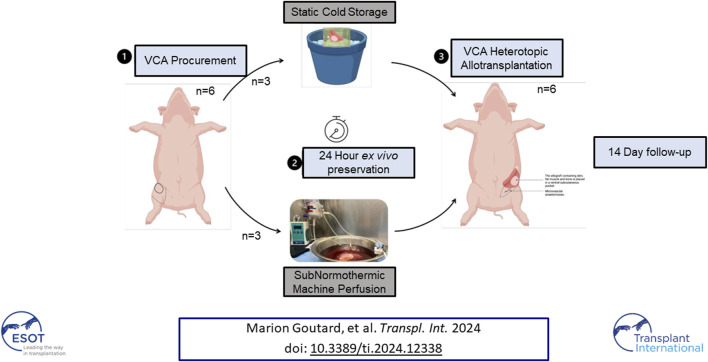

## Introduction

Traumatic injuries (ballistic, high-energy motor vehicle accidents, burns, etc.), congenital diseases, and neoplasia are responsible for severe disfigurements and amputation of functional aesthetic units in patients of all ages. For cases where autologous reconstructive options have been exhausted, vascularized composite allograft (VCA) transplantation offers significant quality of life improvements [1]. Although immense progress has been made in VCA procurement techniques, graft adjustments to the recipient, and post-operative care to achieve optimal results [2, 3], a more effective and longer graft preservation method is needed to make VCA transplantation a routine reconstructive procedure.

tThe method used for *ex vivo* preservation is a key factor in post-transplantation graft function and rejection risks [4–6]. For about half a century now, the combination of University of Wisconsin (UW) solution (or equivalent), an organ bag, and an icebox have been used to store all organs from procurement to transplantation. This method was chosen as a gold standard for its simplicity and cost-effectiveness [7]. However, limitations for this approach are obvious considering that at +4°C tissue metabolism continues at ∼10% of nominal levels, resulting in a maximum preservation time of 4–6 h as the tissue effectively slowly suffocates. Further, cold ischemia causes ongoing injury to the graft, adversely affecting post-operative graft viability and functional outcomes [5, 8, 9].

Hence, the field has been pursuing better technologies to overcome *ex vivo* preservation injury. Machine perfusion (MP) is a promising alternative since it allows dynamic and monitored *ex vivo* organ preservation by supplying allografts with nutrients and oxygen at concentrations tailored to meet specific metabolic needs of the tissue [10, 11]. Oxygenated MP was first optimized in solid organ transplantation and is now clinically used in kidney [12], liver [13], heart [14], and lung [15] transplantations. VCA *ex vivo* perfusion studies are rather recent in the literature and are still mostly confined to animal models. Swine limb models (forelimbs or hindlimbs) are frequently used for VCA preservation studies since they are easy to procure and contain common tissue types required for reconstructive procedures (skin, fat, muscle, nerve, tendon, and bone). Previous studies have suggested the superiority of different machine perfusion protocols in swine limb or myocutaneous flap preservation, when used either *ex vivo* [16–18] or *in situ* after replantation [19–21]. However, to our knowledge, no study reports the outcomes of *ex vivo* perfused VCAs after allotransplantation to assess the impact of immunological incompatibility between donor tissues and the recipient animals, which is a central component of clinical VCA procedures [22].

In this study we used a previously described heterotopic limb allotransplantation model in swine [23] to evaluate the possibility of enhanced *ex vivo* preservation of VCA using MP. Our work relied on 24-h *ex vivo* preservation of VCA using either SCS or subnormothermic machine perfusion (SNMP, +21°C), demonstrating that SNMP preserves VCAs for up to 24 h before allotransplantation, increasing the total viable duration for porcine partial hindlimbs 4–6 times beyond what can be achieved on ice.

## Materials and Methods

### Animals

All animals received humane care in accordance with the National Institutes of Health Guide for the Care and Use of Laboratory Animals. The MGH Institutional Animal Care and Use Committee (IACUC—protocol 2019N000176) and Department of Defense Animal Care and Use Review Office (ACURO) approved the animal protocols used in this study.

### Study Design

**FIGURE 1:**
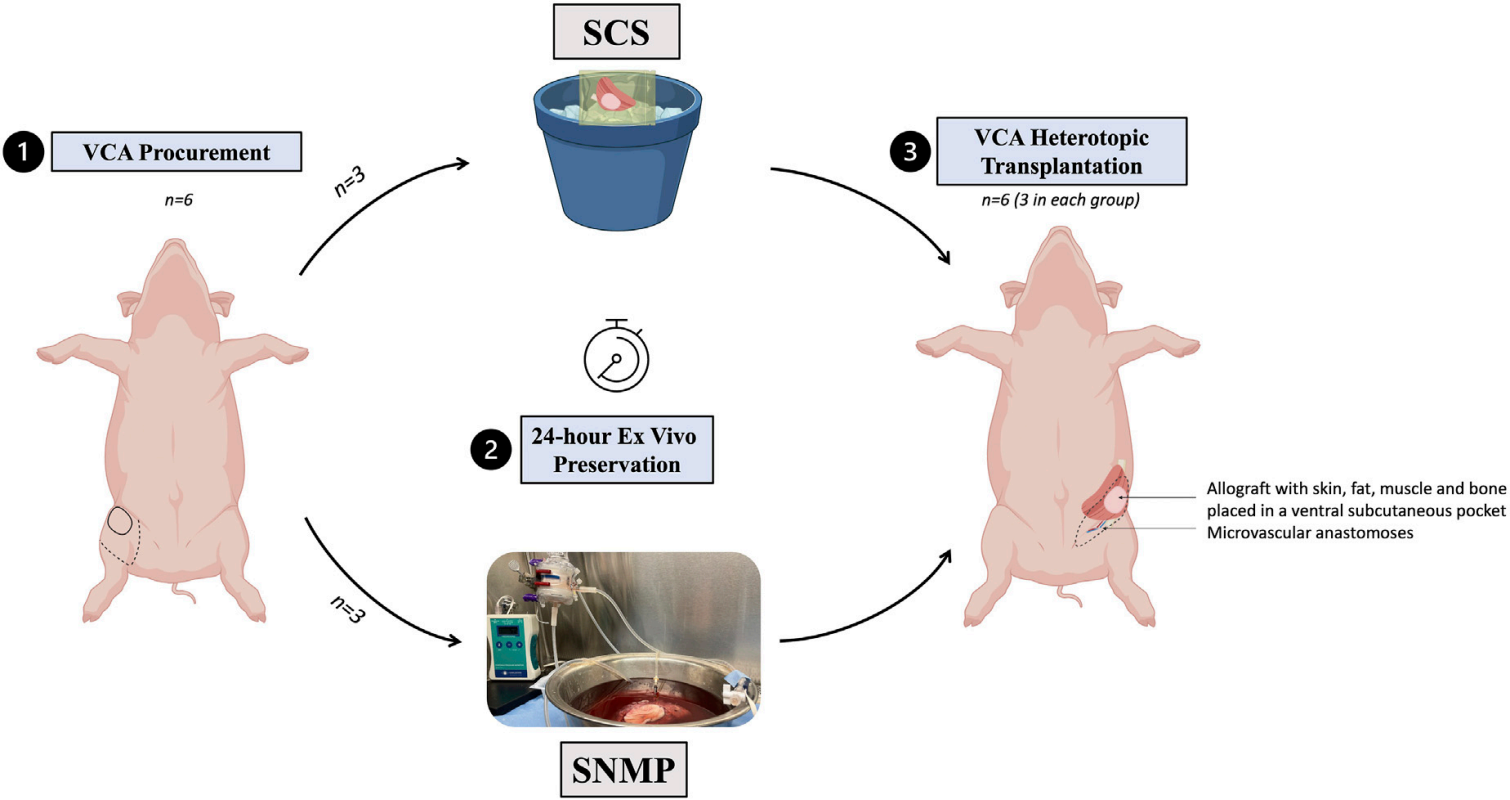
Experimental design of the study. A total of 6 partial hindlimbs were procured, all *ex vivo* preserved for 24 h, 3 using SCS, 3 using SNMP and then allotransplanted in a non-related recipient Yorkshire swine.

This study was designed to compare two *ex vivo* preservation methods in VCA (Figure 1). A total of 12 female outbred Yorkshire pigs were used for all experiments: 6 served as donors for partial hindlimb procurement and 6 were allograft recipients, split equally between the control group of 24-h SCS (group SCS, *n* = 3) and the experimental group of 24-h SNMP (group SNMP, *n* = 3). Recipients and donors were randomly allocated to their group. Donors and recipients were not related in either group. The primary endpoint was the recipient animal survival at end of study (postoperative day 14).

### Partial Hindlimb Procurement

All limb procurement surgeries were non survival procedures under general anesthesia, the animals were humanely euthanized at the end of the surgical procedure according to the American Veterinary Medical Association guidelines for animal euthanasia. Partial hindlimbs consisting of the distal femur, knee-joint, tibia-fibula, surrounding muscles, and a skin paddle vascularized through the femoral artery and vein were procured according to a previously described surgical protocol [23, 24]. An intravenous (IV) injection of 100 IU/kg heparin in the osteomyocutaneous allograft was performed 5 min before ligation of the femoral vessels with 2-0 silk ties just below the inguinal ligament. The femoral artery was then cannulated with a 14 or 16 G angiocatheter (BD Angiocath, Franklin Lakes, NJ, United States) and the partial limb was flushed with 100 mL heparinized saline before being subjected to 24-h *ex vivo* preservation. Weight of the VCA and ischemia times were recorded for each limb.

### Static Cold Storage (SCS)

Osteomyocutaneous flaps subjected to cold storage were flushed with 100 mL +4°C Histidine-tryptophan-ketoglutarate (HTK) solution (Custodiol, Essential Pharmaceuticals LLC, Durham NC, United States) and then immersed in a sterile bag containing 900 mL of cold HTK, transported on ice to a +4°C refrigerator. After 24-h, the allografts were removed from SCS, placed on a wet sterile gauze, and flushed with 100 mL of room temperature saline before being inset in the recipient animal.

### Sub-Normothermic Machine Perfusion (SNMP)

**FIGURE 2:**
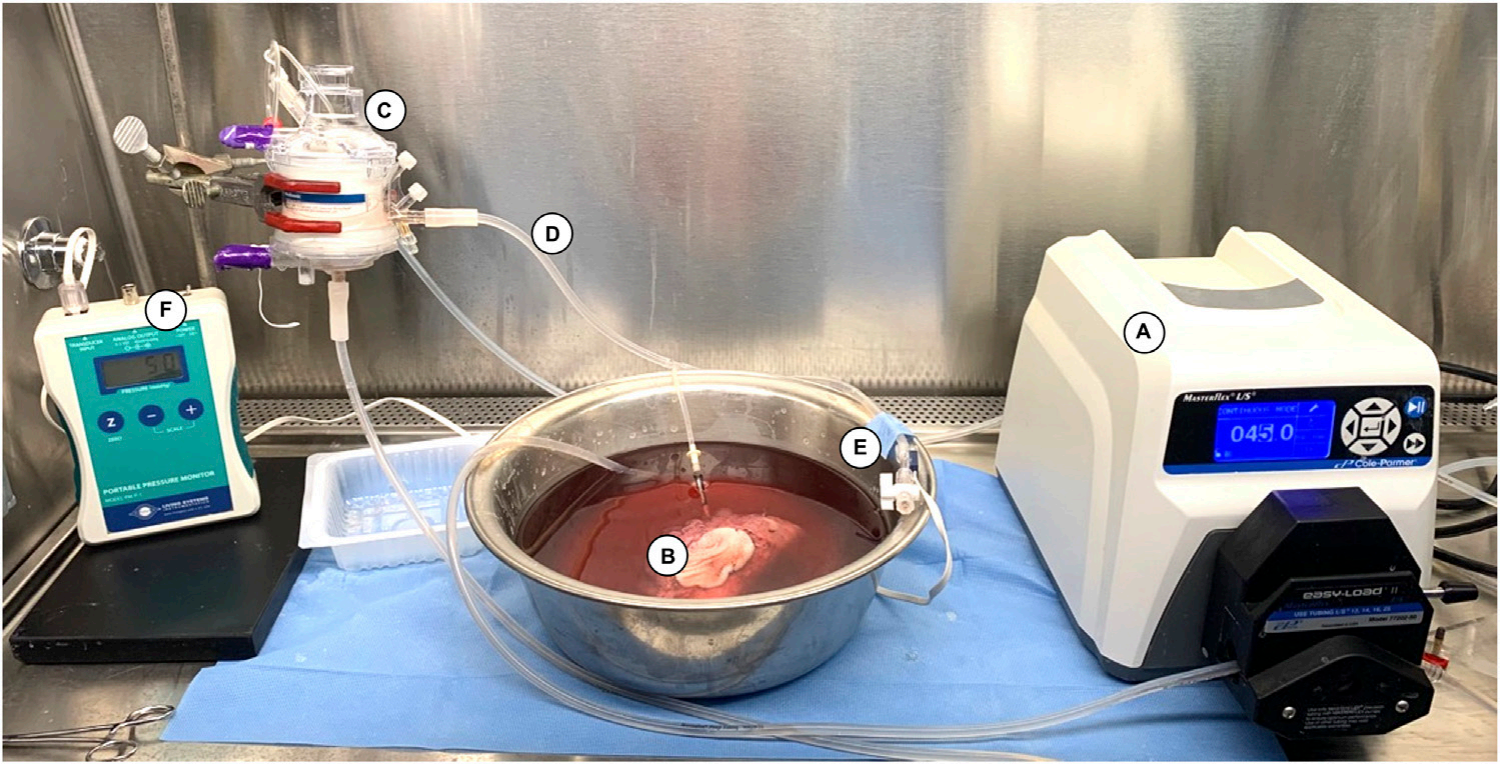
Machine perfusion system used for 24-h SNMP. The system consists of **(A)** a roller pump (07522-20 DRIVE MFLEX L/S 600RPM 115/230, Cole-Parmer, Vernon Hills, IL, United States) delivering a continuous flow of Steen+ solution to **(B)** the VCA through an angiocatheter inserted in the femoral artery, **(C)** a hollow membrane oxygenator (Affinity Pixie™ Oxygenation System, Medtronic, Dublin, Ireland), and **(D)** sterile tubing (Masterflex^®^ L/S Platinum-Cured Silicone Tubing, size 16, Cole Parmer, Vernon Hills, IL, United States). **(E)** A pressure transducer (PT-F, Living Systems Instrumentation, St Albans City, VT) was connected downstream to the inflow catheter and used to record the arterial pressure continuously on **(F)** the monitor (PM-P-1, Living Systems Instrumentation, St Albans City, VT, United States).

Our continuous flow perfusion system was built in a sterile hood in a room at 21°C as displayed in Figure 2. A sterile container was filled with 2 L of modified Steen solution (Steen+) which presents a higher oncotic pressure than that of the original Steen^®^ [25]. The system included a hollow-fiber membrane oxygenator (Affinity Pixie, Medtronic, Dublin, Ireland) Perfusion and a high precision peristaltic pump (Masterflex L/S, Cole Parmer, Vernon Hills, IL, United States). Perfusion was started an hour before VCA perfusion to oxygenate the circulating perfusate and bring the solution temperature to 21°C. A continuous flowrate of 0.5L/min carbogen (5% CO_2_, 95% O_2_) was used to ensure perfusate oxygenation. Before starting the experiment, the solution was tested for pO_2_ and pH with an i-STAT machine (Albott, Princeton, NJ, United States). A sodium bicarbonate 8.4% solution was used as a buffer to maintain a pH above 7.2 if necessary. The allografts were transported on ice to the machine perfusion setup. Once started, the first 50 mL of graft outflow were discarded to avoid accumulation of warm ischemia metabolites in the recirculating perfusate. One-liter (50%) perfusate exchange was performed after 12 h of SNMP. The flowrate was manually adjusted throughout perfusion to maintain an arterial pressure of 40 ±5 mmHg as displayed by continuous pressure monitoring in the closed system (PM-P-1, Living Systems, St. Albans, VT, United States). Pressure, flowrate, and metabolic parameters were measured at 0, 0.5, 1, 2, 3 h, then every 3 h during *ex vivo* perfusion. Circulating pH, pO_2_, pCO_2_, lactate levels, K^+^, Na^+^, HCO_3_^−^ and glucose were tested from outflow and inflow perfusate samples with the iStat machine. Edema was estimated in percentage of weight gain (%) every 6 h (with no decannulation). All allograft manipulations were performed in sterile conditions.

### Allotransplantation of Partial Hindlimbs

#### Surgical Protocol

Recipient pigs were transplanted 24 h after VCA procurement under general anesthesia. The procedure started with isolation of the internal jugular vein through a 10 cm longitudinal incision in the neck and placement of a triple lumen vascular access line (Pressure Injectable Arrowg+ard Blue Plus^®^ Three-Lumen CDC-45703-P1A) for later administration of IV drugs and blood draws. We followed a previously described method for VCA heterotopic transplantation in swine [23, 24]. In brief, an incision was made in the inguinal crease contralateral to the side of procurement and the femoral vessels were isolated and prepared for microvascular transfer. All other vessels outsourcing from the graft were ligated or coagulated to avoid leakage. When the allograft reached 24 h of *ex vivo* preservation, it was removed from the perfusion system or SCS and brought to the recipient site. The allograft was then placed in the ventral subcutaneous pocket and two vascular anastomoses (femoral artery, femoral vein) were performed (Figure 1). After observation of allograft revascularization, the surgical site was closed using absorbable sutures. Recipients were fully recovered within 2 h post-transplantation, singly housed, continuously monitored for the first 6 postoperative hours, and then twice daily. Recipient animals were humanely euthanized in case of graft failure, deteriorated general condition of the animal, or at the end of the study at day 14 post-transplantation. Upon euthanasia, allografts were thoroughly dissected before sampling.

#### Medications

Analgesia: A Fentanyl^®^ patch delivering 50 μg/h was placed on the back on the animal preoperatively and for 72 h 4 mg/kg Carprofen^®^ was administered per os (PO) for the first 72 h and more if needed.

Antibioprophylaxis: 50 mg/kg sulfametoxazol-trimethoprim PO and enrofloxacin 5 mg/kg intramuscular (IM) were given daily for 14 days.

**FIGURE 3:**
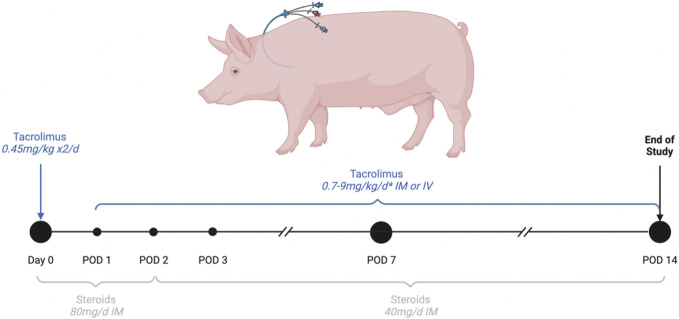
Immunosuppression regimen. Drugs were given IV while the triple lumen central line was functional. *The targeted residual concentration of blood tacrolimus was between 0.4 and 0.6 ng/mL, blood concentration was measured every 2 days and the tacrolimus dose was adjusted to the tacrolimus trough level.

Immunosuppression (Figure 3): Tacrolimus was given once daily IV or IM to ensure a target residual blood concentration between 40 and 60 ng/mL. Steroids IV or IM were started at 80 mg daily and progressively tapered.

#### Postoperative Follow-Up

Recipient animals were monitored continuously during the first 6 h postoperatively. Physical examinations were then performed twice daily during the entire study to monitor signs of allograft rejection, allograft failure, or change in general condition. Blood draws were performed at 6 h, day 1, 3, 7, 10, and 14 post-transplantation for complete blood counts and assessment of potassium, sodium, and lactate blood levels. Tacrolimus residual blood concentration was measured on post-operative day (POD) 2 and every 48 h until levels were stabilized within the targeted therapeutic range (40–60 ng/mL).

### Histology

Muscle biopsies were taken at 0, 6, 12, 18 and 24-h of *ex vivo* preservation and after transplantation at 1-h post-reperfusion and at the end of study. VCA skin punch biopsies (6 mm) were performed at 1-h post-transplantation, POD 1, 3, 7, 10 and 14. All biopsy samples were fixed in formalin, paraffin embedded, sectioned and stained with hematoxylin and eosin (H&E), trichrome, and TUNEL (terminal deoxynucleotidyl transferase dUTP nick end labeling). All biopsies were evaluated by two independent pathologists blinded to the study using the Histology Injury Scoring System (HISS) for hypoxia-induced muscle injury [20], which include evaluation of the muscular architecture, structural myocyte injury and apoptosis. A Pathological Component Scoring System specific for skin-containing VCA and the 2007 Banff classification were used to grade skin components [26, 27].

### Statistics

All statistical analyses were performed using Prism 9 for Mac OSX (GraphPad Software, La Jolla, CA). Population characteristics were compared using a Mann Whitney test. Comparison of categorical data was achieved using non-parametric tests. Mean histology scores were analyzed using a Sidak’s multiple comparison test. *p* values less than 0.05 were considered significant.

## Results

### VCA Characteristics

All procured VCAs had similar baseline characteristics (Table 1). Mean warm ischemia times (before and after *ex vivo* preservation) were comparable between both groups. Perfused VCAs gained an average of 15.5% weight after 24-h SNMP. The weight of cold-stored VCA remained stable with a mean loss of 0.94% weight after 24-h SCS, demonstrating no significant difference with the SNMP group (*p* = 0.10, non-parametric Mann-Whitney test).

**TABLE 1:** Characteristics of VCA and ischemic conditions in each group.

	SCS group (*N* = 3)	SNMP group (*N* = 3)	*p*-Value
VCA weight after procurement (mean ± SD, g)	352.3 ±26.6	350 ± 34	*>0.99*
VCA weight after *ex vivo* preservation (mean ± SD, g)	349 ±25.7	403 ± 58.3	*0.40*
Weight variations with *ex vivo* preservation (mean ± SD, %)	−0.94 ±0.66	+15.5 ± 6.48	*0.10*
Warm ischemia time before preservation (mean ± SD, min)	12.3 ±4.1	14.7 ± 1.5	*0.70*
Warm ischemia time after *ex vivo* preservation^a^ (mean ± SD, min)	105 ±5.5	94.7 ± 10.4	*0.20*

There was no statistical difference between transplanted VCA in both groups (*p* > 0.05 using Mann-Whitney tests).

^a^ Time to perform inset of the allograft and vascular anastomoses.

### Perfusion Outcomes

**FIGURE 4:**
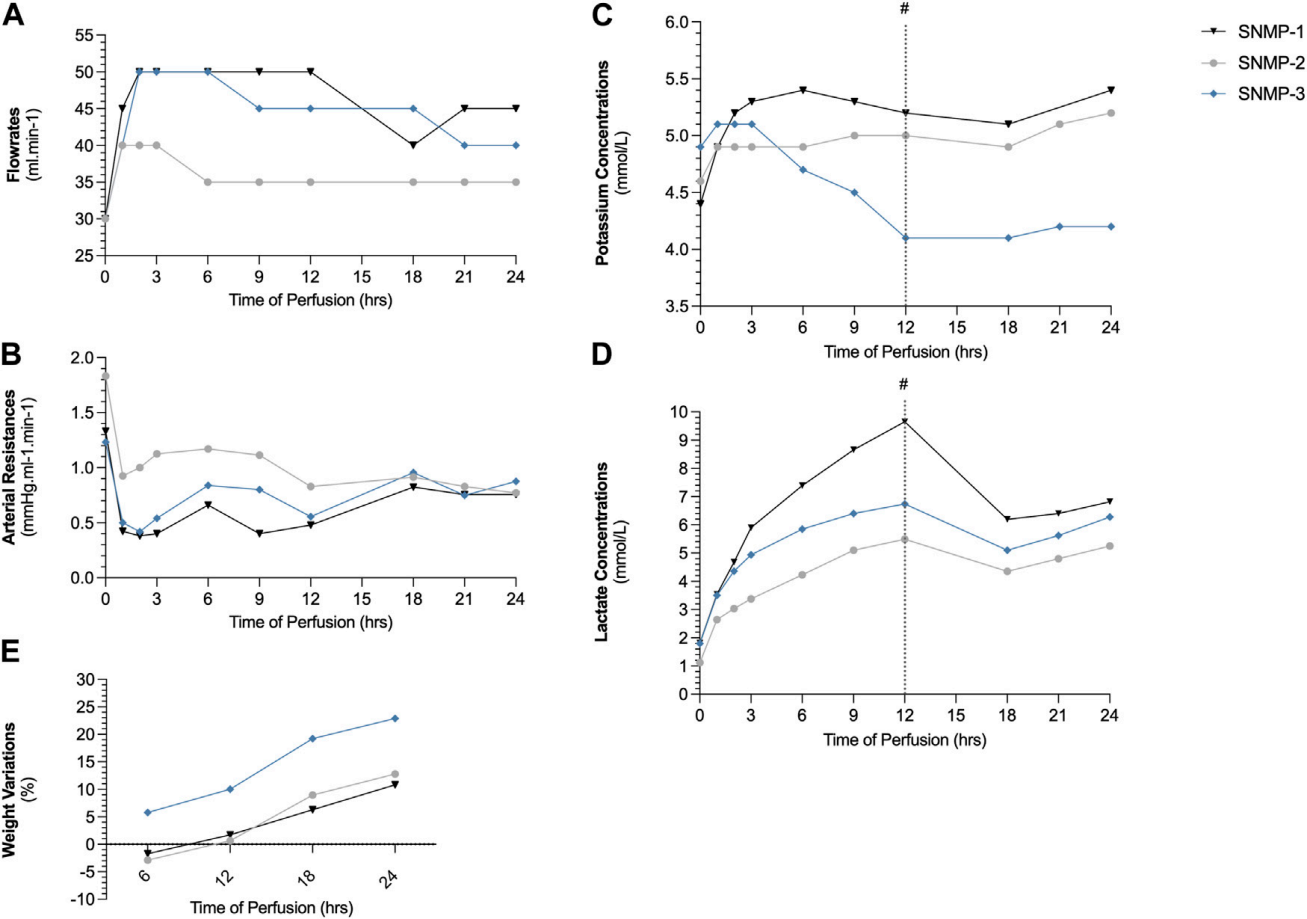
Swine hindlimb perfusion results. Perfusate flowrate was maintained between 35 and 50 mL/min **(A)** to maintain an arterial pressure below 50 mmHg, resulting in arterial resistances between 0.5 and 1 mmHg.ml^−1^.min^−1^ **(B)**. Potassium concentrations **(C)** in the outflow perfusate were within physiologic ranges and lactate concentrations **(D)** decreased thanks to the 1 L perfusate exchange at 12 h (#). Weight gain after 2-h SNMP remained in the range of 10%–22% **(E)**.

Perfusion parameters showed successful preservation by SNMP in all cases (SNMP-1, SNMP-2, SNMP-3). Flowrates (Figure 4A) remained stable in all cases between 35 and 50 mL/min with arterial resistances that approximated 1 mmHg/ml/min at the end of 24-h SNMP (Figure 4B). Weight gain was observed in all perfused VCAs (Figure 4C), staying below 25% after *ex vivo* perfusion. Potassium concentrations were measured within the physiologic range (3.5–5.5 mmol/L) at all timepoints (Figure 4D) and lactate levels were similar in all 3 perfused VCAs (Figure 4E).

### Allotransplantation of Partial Hindlimbs After 24-h *Ex Vivo* Preservation

#### Post-Transplantation Clinical Observations and Survival

**FIGURE 5:**
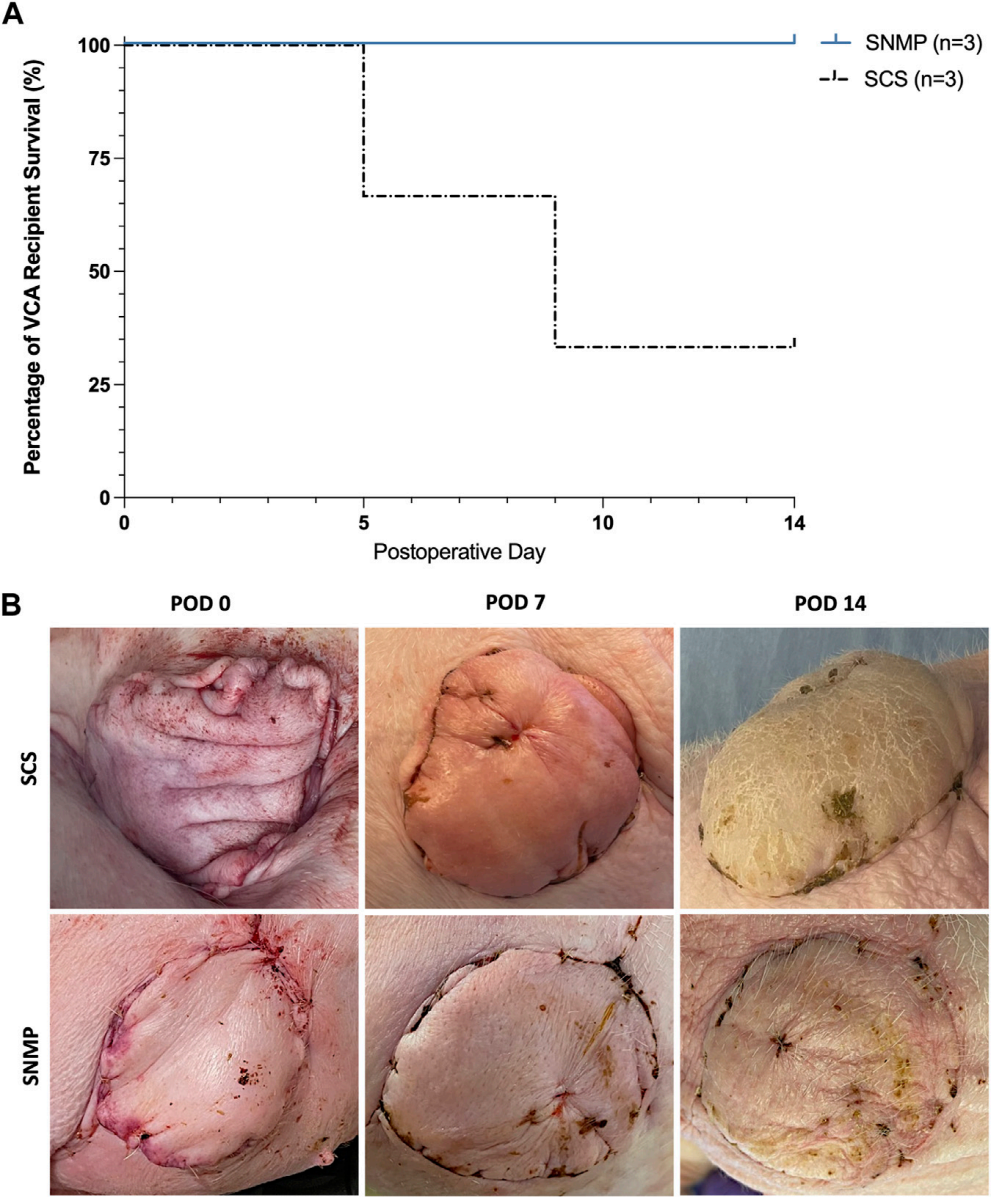
24 h SNMP leads to improved recipient outcomes compared to time-matched SCS. 2 out of 3 recipient animals were euthanized based on veterinary recommendation prior to the end of study in the SCS group, where all animals in the SNMP group were in normal condition **(A)**. Postoperative clinical aspect of the allografts was normal in the SNMP group whereas the SCS recipient that survived showed major allograft edema **(B)**.

All animals from the SNMP group recovered from the allotransplantation surgery within 2 h, and were able to stand, walk and eat normally. None of the 3 recipient pigs of the SNMP group showed sign of pain or distress during follow-up and all remained on study through the end point on POD 14. In the SCS group, 2 out of 3 animals showed deteriorated general condition, loss of appetite and difficulty standing and walking. They were subsequently euthanized at POD 5 and POD 9 after veterinarian examination (Figure 5A). There was no sign of allograft rejection during follow-up in either group under immunosuppressive therapy. In both groups, the VCA skin paddles appeared well vascularized for all recipients throughout the study (Figure 5B). No vascular failure of the VCA occurred during the study.

#### Biological Observations

**FIGURE 6:**
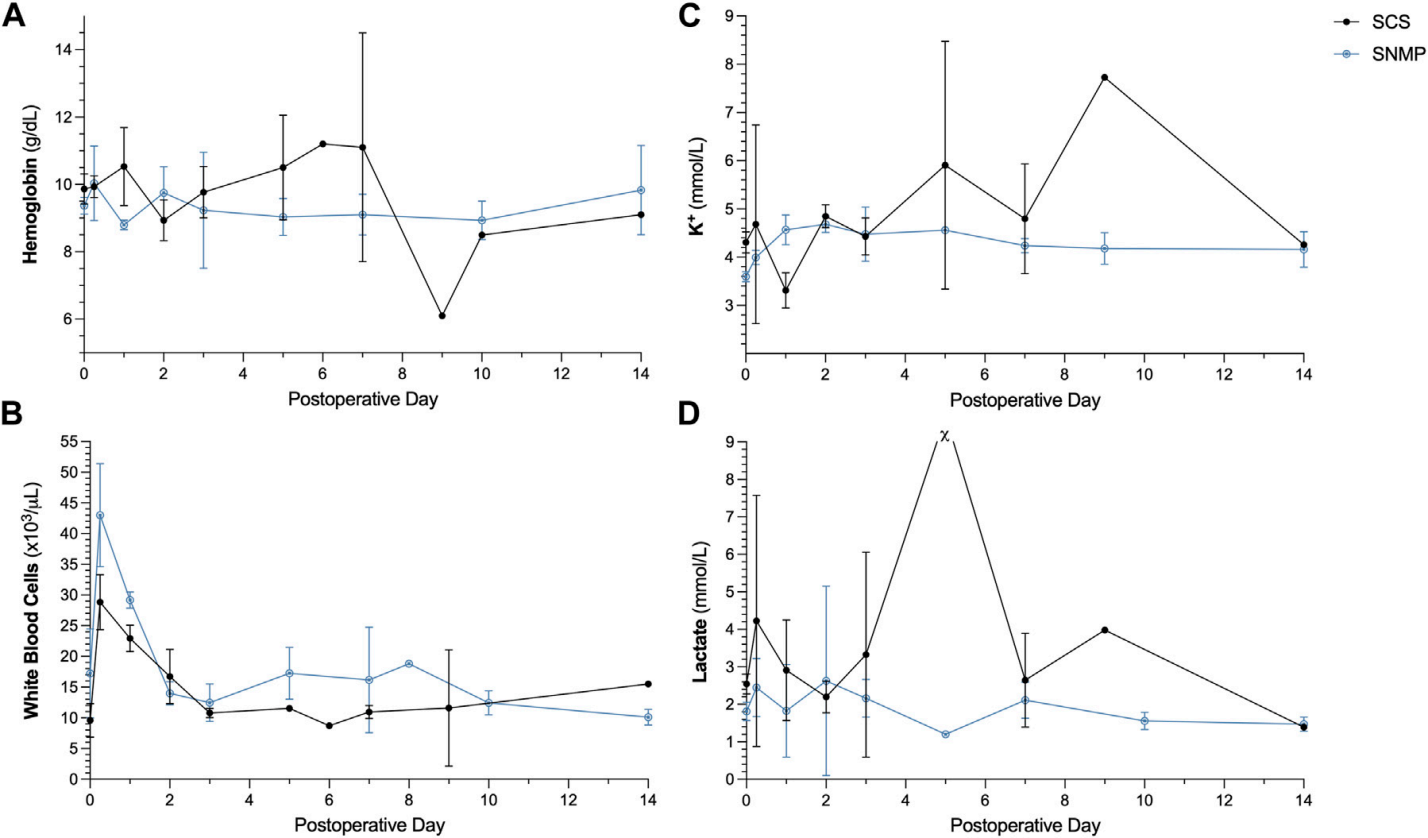
Postoperative blood analyses in recipient animals. Hemoglobin **(A)**, K^+^ **(B)** was similar in both groups. SNMP group displayed increased white blood cell count **(C)** in the immediate postoperative period which resolved spontaneously **(B)**. In the SCS group a high lactate level **(D)** was observed before euthanasia of one the recipient animals on POD 5 (χ for value > 20 mmol/L which is beyond the range of the test).

The SCS group being reduced to only one surviving animal after POD 9, no statistical analysis was performed to compare the biological parameters between groups. In the SNMP group, the recipient pigs had stable hemoglobin level, potassium level and lactate level during follow-up (Figures 6A, C, D). Both groups showed hyperleukocytosis immediately after transplantation that spontaneously resolved by POD 2 (Figure 6B). In the SCS group, abnormal values (low hemoglobin, increased potassium, and lactate levels) were observed on days of euthanasia, POD 5 and 9 (Figures 6A, C, D). Tacrolimus trough levels were in the targeted therapeutic range throughout the follow up period.

#### Macroscopic Observations and Histological Findings at End of Study

All SCS preserved allografts showed massive edema and seroma formation associated with macroscopically visible muscle degeneration even though vascular flow was respected. In this group, allograft skin observations unveiled solely indirect signs of cutaneous suffering: mild epidermolysis and scarce hair regrowth. No ischemic change in skin color was observed. In the SNMP group, allograft skin were unremarkable and muscle aspect was macroscopically normal with good arterial inflow and venous outflow.

**FIGURE 7:**
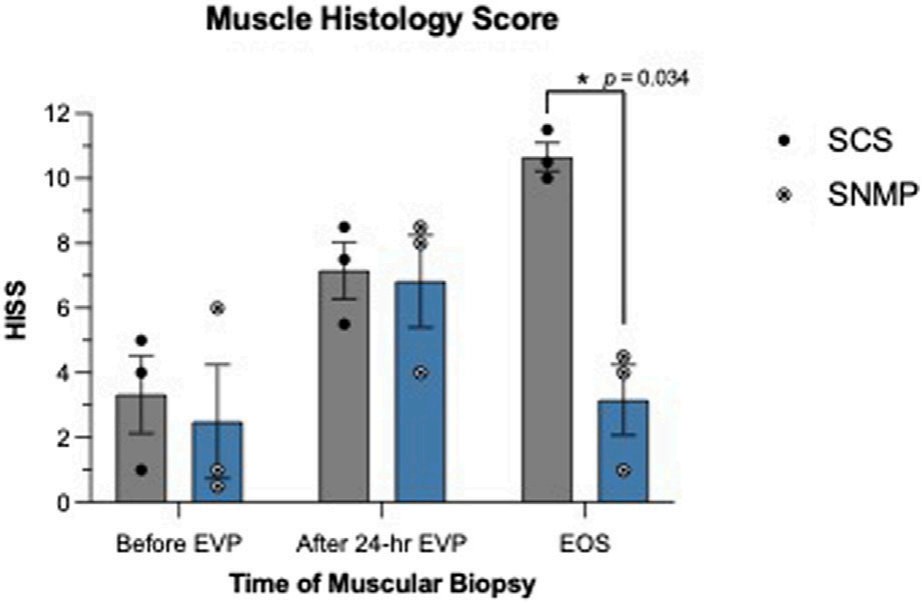
Hypoxia-induced muscle injury score based on the Histology Injury Scoring System performed before and after *ex vivo* preservation. SNMP group was superior to SCS with minimal or no lesions.

**FIGURE 8:**
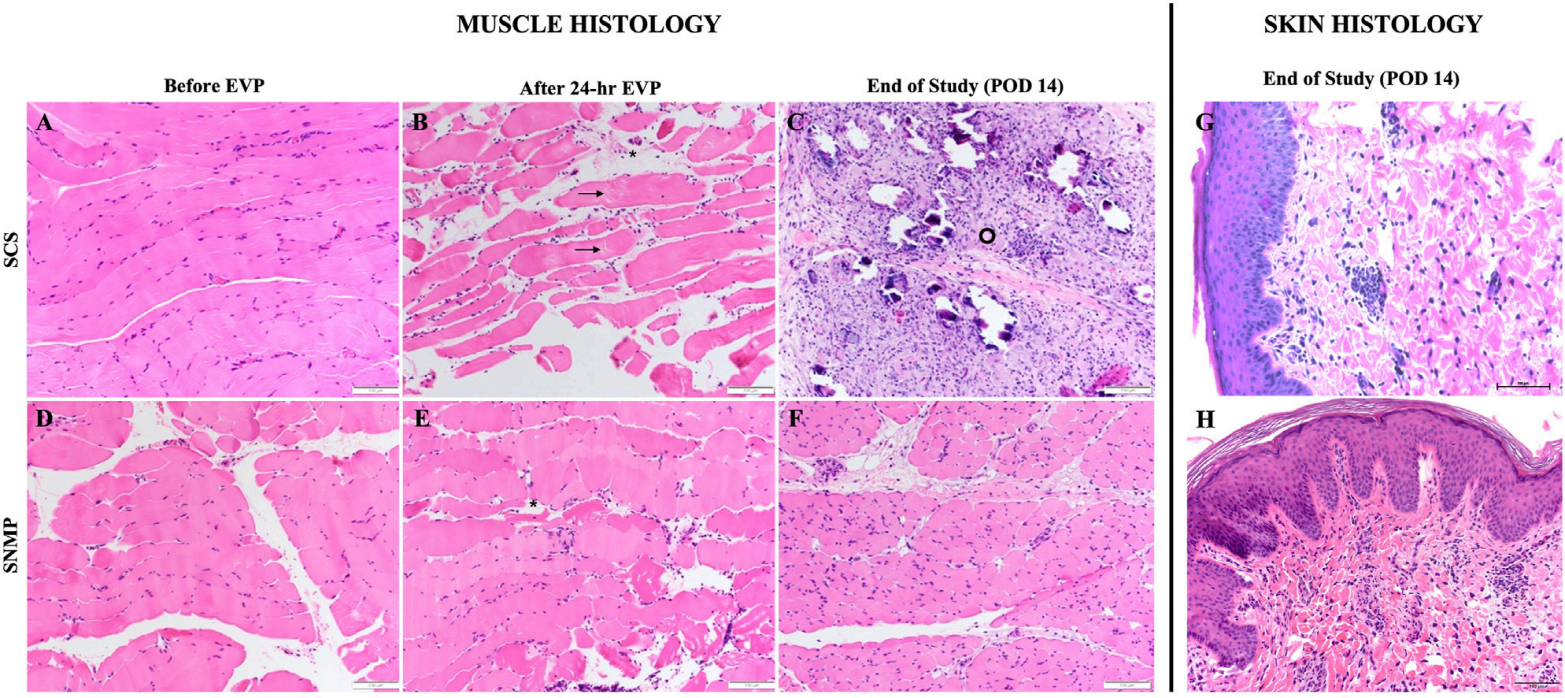
H&E stained sections of VCA biopsies in the SCS group (top row) and the SNMP group (bottom row). In both groups, baseline muscle histology of the VCA after procurement is shown [**(A)** SCS, **(D)** SNMP]. After 24-h SCS, edema between muscle fibers [asterisk] and splitting of sarcoplasm [arrows] were observed **(B)**. 14 days post transplantation, diffuse ischemic changes, myocytes injury, cellular infiltration and increased perimysial connective tissue [ring] were noted **(C)**. Samples from SNMP preservation showed interstitial edema [asterisk] without significant injury **(E)** and restoration of muscle architecture after allotransplantation **(F)**. Skin samples **(G,H)** at end of study showed no rejection (Banff Grade 0) in both groups.

Skin biopsies performed after allotransplantation did not show significant rejection episodes, all samples were classified 0 on the Banff scale by the two blinded pathologists besides the SCS group at the euthanasia time point (Banff grade 1 [1, 2]). Detailed skin histology is displayed in Supplementary Table S1. End of study evaluation of the total histology score on muscle biopsies revealed a significant difference between groups (Figures 7, 8). A mean score of 3.167 was found in the SNMP group, ranking it in the no to minimal degeneration classification of the HISS, whereas a mean of 10.67 was obtained in the SCS group (*p* = 0.034 using a Sidak’s multiple comparison test), marking it as severe degeneration outcome. Histological subscores revealed differences in terms of inflammation, variations in myocytes shape and size, and damaged muscle fibers were always higher/worse in the SCS group (Figure 8; Table 2).

**TABLE 2:** Detailed hypoxic-induced muscle injury score according to the Histology Injury Scoring System.

Hypoxic-induced muscle injury	Time of muscle biopsy	SCS group (*n* = 3)	SNMP group (*n* = 3)
Interstitial edema (median (min, max)) 0. No significant increase 1. Minimal 2. Intermediate 3. Severe/diffuse	Before EVP	0 (0, 0)	0 (0, 3)
	After 24-h EVP	3 (1, 3)	2 (2, 3)
	EOS	**0 (0, 2)**	**0 (0, 1)**
Inflammation [median (min, max)] 0. Not significant 1. Minimal 2. Intermediate 3. Diffuse	Before EVP	0 (0, 1)	0 (0, 0)
	After 24-h EVP	0 (0, 0)	0 (0, 1)
	EOS	**3 (2, 3)**	**0 (0, 0)**
Variation in myocytes shape and size (median (min, max)) 0. Homogeneous 1. Mild heterogeneous 2. Intermediate heterogeneous 3. Severe heterogeneous	Before EVP	0 (0, 1)	0 (0, 1)
	After 24-h EVP	1 (1, 3)	2 (1, 3)
	EOS	**3 (3, 3)**	**0 (0, 1)**
Damaged muscle fibers (median (min, max))	Before EVP	1 (0, 1)	0 (0, 2)
0. 0–5 myocytes/10 hpf (×20 magnification)	After 24-h EVP	1 (1, 3)	2 (2, 3)
1. 6–20 myocytes/10 hpf (×20 magnification)	EOS	**3 (3, 3)**	**0 (0, 0)**
2. 21–50 myocytes/10 hpf (×20 magnification)
3. >51 myocytes/10 hpf (×20 magnification)

EVP, *Ex Vivo* Preservation; EOS, End of the Study.

The bold values correspond to the values of most interest (End of the study time points).

## Discussion

After several breakthrough clinical trials in solid organ preservation, machine perfusion is permeating the field of VCA, aiming to enhance preservation duration and allograft viability [7]. In this study, we demonstrated the superiority of preserving VCAs for 24 h with a SNMP protocol compared to conventional time-matched SCS in a porcine heterotopic limb allotransplantation model.

In previous reports, *in vivo* viability of VCA after machine perfusion preservation was assessed using replantation models with follow-up durations ranging from 12 h to 7 days. In 2017, Kueckelhaus et al. were to the first to replant porcine forelimbs after 12 h of hypothermic perfusion [16]. They observed reduced ischemic injuries in perfused limbs compared to standard 4-h SCS, thus extending VCA preservation up to 3 times longer than the gold standard. Two years later, the same team extended the perfusion time to 24 h and validated *in vivo* viability with a similar replantation model [19]. More recently, Kruit et al. replanted forelimbs after 18-h SNMP preservation and followed the recipient pig for 12 h to assess muscle function and histology compared to 4-h SCS preservation [20]. Our findings were in line with these results, as we were able to preserve VCAs for 24 h in subnormothermic conditions with stable arterial resistances, moderate edema, and outflow potassium and lactate concentrations remaining in physiological values. Additionally, pigs that received perfused VCAs showed good clinical tolerance and did not suffer any systemic reaction, infection, or acute allograft rejection. In contrast, 2 out of 3 SCS control pigs developed poor general condition most likely due to graft failure (inflammation, rhabdomyolysis as suggested by high potassium levels). The lactate spike found in one of the SCS animals was considered resulting from hepatic and multi-organ failure resulting from graft failure. Pain management was considered optimal by experienced veterinarians. Complete necropsies were performed on all animals euthanized before POD14, and no alternative conditions such as sepsis or disseminated intravascular coagulation were found. Despite the lack statistical significance, qualitative assessment of muscle histology in the SCS group showed major muscle degeneration, supporting the hypothesis of graft failure leading to the poor general condition of the recipient animals. However, graft failure was not clinically obvious given that there was no direct sign of graft ischemia and graft degeneration was concluded upon euthanasia dissection. Hence, delayed VCA failure can insidiously occur as late as POD9 with no or minimal skin lesions. This phenomenon was previously observed in rat hind limb transplantations after not 24 but 48 h of SCS. Interestingly, histological analyses of muscle biopsies showed no difference between SNMP and SCS immediately after *ex vivo* preservation but revealed a statistical difference at the end of study in favor of SNMP, underlining the importance of *in vivo* analysis of the allograft in a longer follow up period than previously described. Our hypothesis is that reperfusion injuries slowly deteriorate muscular cells leading to extended myocyte apoptosis and microvascular damage, viciously killing the muscle tissue and releasing necrosis marker in the systemic circulation although those markers have not been investigated in this study.

Protocols based on Subnormothermic perfusion appear to strike a balance in temperature. Hypothermic machine perfusion has generally led to satisfactory outcomes in VCA preservation [16, 20, 28] without additional oxygen carriers [28]. However, cooling VCAs down to 8°C–10°C appears to result in vascular injuries and subsequent higher interstitial edema (considered clinical revelation of vascular leakage induced by endothelial damage during *ex vivo* storage), especially after 24 h of *ex vivo* perfusion. Krezdorn et al. reported an average weight gain of +41% of after 24 h of hypothermic perfusion [19]. Similarly, Kruit et al. reported +18.6% of weight variation after 18 h of hypothermic perfusion [20], while we were able to limit edema formation to an average of +15% weight for all limbs after 24 h of SNMP. These results are also in line with previous studies on SNMP organ preservation [25, 29], where SNMP was used as an organ reconditioning phase after an ischemic period and prior to transplantation. However, we did not directly compare SNMP with different temperatures in this study. A direct comparison utilizing the same protocols and models are needed to draw further conclusions and develop a mechanistic understanding of how perfusion temperature effects this very complex tissue. Low-temperature preservation has the potential to mitigate cell metabolism, therefore suggesting longer preservation durations [30]. Subzero preservation techniques seem to be promising alternatives for multi-day preservation of solid organs [31–35], but solid evidence still needs to be demonstrated in VCA. In the realm of Normothermic Machine Perfusion (NMP), Fahradyan et al. [36] demonstrated 12-h preservation of porcine VCAs with preserved weight and muscle contractility. They suggested that extending normothermic red blood cell perfusion to 24 h could be feasible, albeit with limited significant evidence *versus* SCS controls. A pioneering work from Werner et al. [37] in fresh human arms demonstrated encouraging results of 24-h near-normothermic (30°C–33°C) perfusion. This sub-physiologic protocol however imposed using a blood-derived perfusate for adequate oxygen transportation. In contrast, the use of subnormothermic “room” temperatures (20°C–25°C) in machine perfusion offers several advantages. First, it simplifies the perfusion device setup, eliminating the need for a heating or cooling system [29, 38]. Second, it allows clinicians to reach a balance between oxygen needs and subdued tissue metabolism resulting in perfusate simplifications [28]. Perfusates that require red blood cells or oxygen carriers that are essential to normothermic “physiological” conditions are no longer needed to ensure adequate oxygenation of the composite tissue at +20°C, thus eliminating the potential immunogenicity of such additional components [39].

In our experiments, all limbs were perfused with a modified acellular Steen solution [25] to minimize edema. Fine-tuned control to maintain a low-pressure circuit (around 30 mmHg) was also needed to mitigate edema—likely due to excessive endothelial shear stress at high pressures - using a continuous flow perfusion pump. Whether non-linear or continuous flow provides better outcomes in VCA machine perfusion still remains unclear [40, 41]. Our previous work demonstrated that non-linear flow has some advantages over continuous flow with improved endothelial function and decreased ischemic injury, although the differences were limited [41]. In the present study we utilized continuous flow for practical reasons.

Allotransplantation is only rarely performed in machine perfusion studies, likely due to cost-related issues. To our knowledge, this study is the first to report allotransplantation of perfused VCA in swine with a 2-week follow up. Our laboratory has been extensively working on VCA in miniature swine to implement new tolerance induction protocols in VCA, rising our expertise in immunosuppressants in swine [42, 43]. Consequently, our experience led us to use a bitherapy made of tacrolimus and steroids. As a matter of fact, on short term studies, the immunogenic risk is mostly confined to acute rejection episodes, which are usually mitigated with high-dose steroids [44].

A major limitation of this work was the small sample size (*n* = 3 per group). Performing allotransplantation instead of replantation to approximate clinical settings subsequently doubled the number of swine needed, thereby limiting the group sizes. While this limits the power of the statistical analysis, leading to increased risk of type-II errors, the SNMP group notably showed clear improved results including a higher recipient survival rate after transplantation, which in our opinion provided additional evidence to a growing VCA perfusion literature. We were also unable to assess limb function after transplantation with this surgical model. However, based on the positive results of this study, our SNMP protocol can be used in other swine VCA models to achieve this goal. Thus, one could argue that 24-h SCS is far more than what is usually the accepted limit for cold ischemia time in human clinical setting for VCA. Although, no studies have proven that swine VCA respond exactly as human tissues. Our previous experience in rat VCA preservation studies showed that rat VCA graft survival was similar after 6 and 24 h of SCS [25]. If graft injuries were evidently expected after 24 h SCS, it served as a negative control to our 24-h SNMP protocol. Since this work mainly aimed at testing the compatibility of 24-h SNMP with successful VCA allotransplantation, another limitation is the lack of focus on nerve histology and nerve conduction assessment, which should be specifically addressed in further studies.

Overall, we showed that a simplified SNMP protocol allowed 24-h VCA preservation in a swine allotransplantation model with confirmed tissue viability until 14 days after allotransplantation. Further studies should focus on assessing neuromuscular function in a long-term follow-up to match the clinical setting.

## Data Availability

The raw data supporting the conclusion of this article will be made available by the authors, without undue reservation.

## Abbreviations

ACURO, Animal Care and Use Review Office; EOS, End of the Study (euthanasia); H&E, Hematoxylin and Eosin; HISS, Histology Injury Scoring System; HTK, Histidine-tryptophan-ketoglutarate (solution); IACUC, Institutional Animal Care and Use Committee; MP, Machine Perfusion; POD, Postoperative Day; SCS, Static Cold Storage; SNMP, Subnormothermic Machine Perfusion; TUNEL, terminal deoxynucleotidyl transferase dUTP nick end labeling; UW, University of Wisconsin (solution); VCA, Vascularized Composite Allograft.
