# Supplementary material for: Machine Perfusion Enables 24-h Preservation of Vascularized Composite Allografts in a Swine Model of Allotransplantation
Source: Transpl Int. 2024 May 15;37:12338. doi: 10.3389/ti.2024.12338 (PMC11133529; doi:10.3389/ti.2024.12338)
Supplement: Supplementary file 1 [file Table1.docx]

**Supplementary Appendix** :

**Supplementary Table 1**: Detailed skin histology scoring according to the Pathological Component Scoring System ([Rosales et al. (2016)]

| **PATHOLOGY SKIN COMPONENT SCORE** | **Time of skin biopsy** | **SCS group (n=3)** | **SNMP group (n=3)** |
| --- | --- | --- | --- |
| ***Perivascular cells/dermal vessels***  ***(median (min, max))*** | *Transplantation +1h* | 0 (0, 0) | 0 (0, 0) |
|  | *End of the study* | 1 (1,2) | 1 (0, 1) |
| ***Perivascular dermal infiltrate area***  ***(median (min, max))*** | *Transplantation +1h* | 0 (0, 0) | 0 (0, 0) |
|  | *End of the study* | 1 (0,2) | 0 (0, 1) |
| ***Luminal leukocytes/capillary or venule***  ***(median (min, max))*** | *Transplantation +1h* | 0 (0, 0) | 0 (0, 1) |
|  | *End of the study* | 2 (0, 3) | 1 (1, 1) |
| ***Epidermal infiltrate***  ***(median (min, max))*** | *Transplantation +1h* | 0 (0, 0) | 0 (0, 1) |
|  | *End of the study* | 0 (0, 0) | 0 (0,0) |
| ***Epidermal apoptosis or necrosis***  ***(median (min, max))*** | *Transplantation +1h* | 0 (0, 0) | 0 (0, 0) |
|  | *End of the study* | 0 (0, 0) | 0 (0, 0) |
| ***Endarteritis***  ***(median (min, max))*** | *Transplantation +1h* | 0 (0, 0) | 0 (0, 0) |
|  | *End of the study* | 0 (0, 2) | 0 (0, 0) |
| ***Chronic allograft vasculopathy***  ***(median (min, max))*** | *Transplantation +1h* | 0 (0, 0) | 0 (0, 0) |
|  | *End of the study* | 0 (0, 0) | 0 (0, 0) |
| ***Banff Grade***  ***(median (min, max))*** | *Transplantation +1h* | 0 (0, 0) | 0 (0, 0) |
|  | *End of the study* | 1 (1, 2) | 0 (0, 0) |
